# Genomics Reveals a Unique Clone of *Burkholderia cenocepacia* Harboring an Actively Excising Novel Genomic Island

**DOI:** 10.3389/fmicb.2017.00590

**Published:** 2017-04-06

**Authors:** Prashant P. Patil, Swapna Mali, Samriti Midha, Vikas Gautam, Lona Dash, Sunil Kumar, Jayanthi Shastri, Lipika Singhal, Prabhu B. Patil

**Affiliations:** ^1^Bacterial Genomics and Evolution Laboratory, CSIR-Institute of Microbial TechnologyChandigarh, India; ^2^Department of Microbiology, Topiwala National Medical College & BYL Nair Charitable HospitalMumbai, India; ^3^Department of Medical Microbiology, Postgraduate Institute of Medical Education and ResearchChandigarh, India

**Keywords:** whole genome sequencing, *Burkholderia cepacia* complex, Genomic islands, horizontal gene transfer, phylogenomics, genomics and evolution, comparative genomics

## Abstract

*Burkholderia cenocepacia* is a clinically dominant form among the other virulent species of *Burkholderia cepacia* complex (Bcc). In the present study, we sequenced and analyzed the genomes of seven nosocomial Bcc isolates, five of which were isolated from the bloodstream infections and two isolates were recovered from the hospital setting during the surveillance. Genome-based species identification of the Bcc isolates using a type strain explicitly identified the species as *B. cenocepacia.* Moreover, single nucleotide polymorphism analysis revealed that the six isolates were clonal and phylogenetically distinct from the other *B. cenocepacia*. Comparative genomics distinctly revealed the larger genome size of six clonal isolates as well as the presence of a novel 107 kb genomic island named as BcenGI15, which encodes putative pathogenicity-associated genes. We have shown that the BcenGI15 has an ability to actively excise from the genome and forming an extrachromosomal circular form suggesting its mobile nature. Surprisingly, a homolog of BcenGI15 was also present in the genome of a clinical isolate named *Burkholderia pseudomallei* strain EY1. This novel genetic element is present only in the variants of *B. cenocepacia* and *B. pseudomallei* isolates suggesting its interspecies existence in the main pathogenic species of the genus *Burkholderia*. In conclusion, the whole genome analysis of the genomically distinct *B. cenocepacia* clinical isolates has advanced our understanding of the epidemiology and evolution of this important nosocomial pathogen as well as its relatives.

## Introduction

*Burkholderia cepacia* complex (Bcc) consists of Gram-negative, oxidase-positive, non-fermenting saprophytic bacilli belonging to the *Burkholderiaceae* family comprising 20 taxonomically valid species (De Smet et al., 2015; Estrada-de los Santos et al., 2015). Members of the Bcc have emerged as important nosocomial pathogens worldwide due to their high intrinsic resistance to most of the antibiotics and antiseptics as well as their survival ability in the limited nutrition environment for a prolonged period (Gautam et al., 2011; Sousa et al., 2011). The outbreaks by members of Bcc have been reported in the intensive care units (Lee, 2008), dialysis patients (Romero-Gomez et al., 2008), and transplant patients (Boszczowski et al., 2014) worldwide. Members of the Bcc are most common contaminants of many pharmaceutical end products and pharmaceutical manufacturing environments (Torbeck et al., 2011). *B. cenocepacia* is the dominant and virulent species of Bcc all over the world, which is responsible for nosocomial infections and is an important pathogen in cystic fibrosis patients (Mahenthiralingam et al., 2005; Drevinek and Mahenthiralingam, 2010; Vial et al., 2011; Parkins and Floto, 2015).

The evolution of bacteria is an outcome of multiple genetic mechanisms occurring inside the cell including horizontal gene transfer (HGT) (Ochman et al., 2000; Gogarten and Townsend, 2005; Wiedenbeck and Cohan, 2011; Polz et al., 2013). HGT shapes the genome of bacteria by allowing rapid acquisition of novel adaptive functions that can have enormous impact on the adaptation and evolution of bacteria (Frost et al., 2005; Gogarten and Townsend, 2005). Genomic islands (GEI), which are the chromosomal segments acquired through HGT are capable of encoding functions that can be advantageous for the host. (Dobrindt et al., 2004; Gogarten and Townsend, 2005; Juhas et al., 2009). Moreover, some GEI encodes their machinery for the excision, conjugative transfer, and the integration. This class of GEI is classified as the Integrative and Conjugative Element (ICE) (Wozniak and Waldor, 2010; Johnson and Grossman, 2015; Novick and Ram, 2016). There is another interesting class of GEI, which possesses autonomous machinery for integration and excision but does not encode conjugation machinery or encodes only few conjugation modules. That class is categorized as the Integrative Mobilizable Element (IME) (Bellanger et al., 2014). However, the IMEs are not self-mobilizable but can be mobilized with the help of conjugation machinery of the helper elements such as conjugative plasmids or other ICEs (Guglielmini et al., 2011). These ICEs and IMEs are rapidly growing classes of the mosaic mobile genetic elements (MGE), which are present in both Gram-negative and Gram-positive bacteria, and can play important roles in the bacterial genome plasticity or instability (Wozniak and Waldor, 2010; Darmon and Leach, 2014). In general, GEIs play a major role in the adaptive evolution of bacteria by dissemination of antibiotic resistance genes and virulence factors leading to the generation of superbugs as well as the dispersal of catabolic genes in the environmental, symbiotic, and commensal bacteria forming new metabolic pathways (van der Meer and Sentchilo, 2003).

Genomics is revolutionizing the field of clinical microbiology. Improvement of the existing technologies and the reduced cost of sequencing are enabling rapid and whole genome-based studies on a large number of isolates (Didelot et al., 2012; Bertelli and Greub, 2013). Moreover, the molecular epidemiological studies have shifted effectively from studying a few housekeeping genes to investigate the whole genome dynamics in each coding region (Maiden et al., 2013). Genome-based taxonomic and phylogenetic analysis are emerging significantly as the modern standards of species identification, and thereby enabling accurate genotyping of nosocomial isolates with high accuracy (Richter and Rosselló-Móra, 2009; Leopold et al., 2014). Similarly, the large-scale comparative genomics is allowing us to identify bacterial isolates involved in an outbreak or evolved in a hospital setting rapidly by acquiring the novel genes through the extensive HGT events (Mellmann et al., 2011; Juhas, 2015).

In the present study, we performed whole genome sequencing and analysis of the nosocomial *B. cenocepacia* isolates obtained from a hospital in city Mumbai, India. This study revealed the existence of a genomically distinct clone of *B. cenocepacia* in a hospital setting. The study also provided valuable insights into a newly identified GEI through the comparative genomics approach. Moreover, the results obtained in this study also recognized the role of HGT events in shaping the genomic diversity and the emergence of variant nosocomial isolates.

## Materials and Methods

### Isolation and Identification of Bcc

Blood cultures were collected aseptically in brain heart infusion broth (BHI) with 0.025% sodium polyanethol sulfonate (SPS) and were processed by the conventional method. Five blood culture isolates were obtained from patients admitted to a Pediatric intensive care unit (PICU), Neonatal intensive care unit (NICU) and Pediatric ward (PW) of the Topiwala National Medical College & BYL Nair Charitable Hospital, Mumbai during the year 2012–2013. Two Bcc isolates were obtained from the upper surface of the rubber cap of multi-dose amikacin vials during the surveillance of hospital environment (swab culture). The non-fermenting Gram-negative bacilli (NFGNB) isolates were identified using Bcc selective media (*B. cepacia* selective agar, BCSA) and biochemical tests, which were further confirmed as Bcc by matrix-assisted laser desorption ionization–time of flight (MALDI–TOF). Ethics approval and patient’s written consent was not required as it was a part of routine clinical testing. Permission for whole genome sequencing (WGS) was approved by Institutional Biosafety Committee of Institute of Microbial Technology (IMTECH), Chandigarh (IMTECH/IBSC/2013/11).

### Genome Sequencing, Assembly and Annotation

Bacterial strains were grown on Mueller-Hinton agar plates from frozen stocks, and genomic DNA were isolated using ZR Fungal/Bacterial DNA MiniPrep Kit (Zymo Research Corporation, Orange, CA, USA), which were further quantified by using Qubit 2.0 Fluorometer (Invitrogen, Carlsbad, CA, USA). Illumina sequencing libraries were prepared by using Nextera XT sample preparation kit (Illumina, Inc., San Diego, CA, USA) with dual indexing. Sequencing libraries were normalized and sequenced on in-house Illumina Miseq (Illumina, Inc., San Diego, CA, USA) platform. All isolates except BC-41 were sequenced by using 250 bp × 2 bp read length in single multiplexed run and BC-41 with 300 bp × 2 bp read length in a separate multiplexed run. Illumina reads were *de novo* assembled using CLC Genomics Workbench 6.5.1 (CLC Bio-Qiagen, Aarhus, Denmark) with minimum contig length 500 bp and otherwise by default parameters. The genomes were submitted to NCBI GenBank database and annotated using NCBI Prokaryotic Genome Annotation Pipeline (Tatusova et al., 2013).

### Genome-Based Species Identification of Bcc Isolates

Average nucleotide identity (ANI) was calculated using JSpecies (Richter and Rosselló-Móra, 2009) and digital DNA–DNA hybridization (dDDH) was calculated using web tool GGDC 2.0^1^. ANI and dDDH values between nosocomial isolates of Bcc and the type strain of *B. cenocepacia* and *B. cepacia* were calculated. ANI heat map was constructed using GENE-E software^2^.

### Single Nucleotide Polymorphism (SNP)-Based Phylogenetic Analysis

Single nucleotide polymorphism (SNP) analysis was carried out using online web server CSI phylogeny 1.1a (Kaas et al., 2014)^3^ available at Centre for Genomic Epidemiology^4^. This algorithm uses freely available programs for read mapping, high-quality SNP calling, and construction of phylogeny tree. First raw reads of nosocomial isolates were mapped to IPCU-A isolate by using Burrows-Wheeler Aligner (BWA) version 0.7.2 (Li and Durbin, 2009). Depth at mapped position was calculated using BEDTools version 2.16.2 (Quinlan and Hall, 2010). SNP calling was carried out using mpileup package of SAMTools version 0.1.18 (Li et al., 2009). Those SNPs were filtered out, which had average depth at SNP position below 10X or 10% of average, less than 10 bp average distance between two SNPs, and quality score less than 30. Also, all insertions and deletions were excluded. SNP-based phylogenetic tree was visualized using MEGA6 (Tamura et al., 2013).

### Comparative Genomics

Genomes of nosocomial isolates were compared with the publically available genomes of *B. cenocepacia* by using BLAST-based tool BRIG version 0.95 (Alikhan et al., 2011) and aligned with reference genome by using MAUVE version 2.4.0 (Darling et al., 2004). Genomic regions were compared using Artemis Comparison Tool (ACT) (Carver et al., 2005). Gene cluster organization and their comparisons were visualized using Easyfig version 2.1 (Sullivan et al., 2011).

### *In silico* Characterization of Genes Encoded by BcenGI15

The sequences of BcenGI15 were extracted from respective genomes and were annotated using RAST server^5^ (Aziz et al., 2008). ORF predicted by RAST were further subjected to BLASTP with non-redundant protein database (nr) to determine putative function.

### Analysis of Insertions and Detection of Extrachromosomal Circular Form of BcenGI15

Primer pairs P1/P2 and P3/P4 that cover *attL* and *attR* sites, respectively, were designed and used to confirm genomic insertion of BcenGI15. Primer pair P2/P3 oriented toward left and right chromosomal BcenGI15 junctions were used to detect the episomal form of BcenGI15 containing *attP* site. All specific primers used in PCR are listed in Supplementary Table 1. Genomic DNA was isolated from an overnight grown culture in LB at 37°C using ZR Fungal/Bacterial DNA MiniPrep Kit (Zymo Research Corporation, Orange, CA, USA) and used as a template. PCR was performed using Phusion polymerase (Thermo Fisher Scientific Inc., Waltham, MA, USA) according to manufacturer’s instructions. The cycling conditions were used as follows: initial denaturation (98°C, 30 s); 30 cycles consisting of denaturation (98°C, 10 s), primer annealing (estimated primer annealing temperature, 20 s), and extension (72°C, 2 min); followed by a final extension (72°C, 10 min). The second round of PCR amplification was carried out as we got a weak band in excised episomal form of BcenGI15 using same PCR primers. PCR products were visualized using 1% agarose gel stained with Good View (SBS Genetech, China). PCR products containing *attL, attR* and *attP* sites were purified using ExoSAP-IT PCR Clean-up kit (Affymetrix, Cleveland, OH, USA) and purified PCR products were sequenced using Sanger sequencer ABI PRISM 3130 Genetic Analyser (Thermo Fisher Scientific Company, Waltham, MA, USA).

### Quantitative PCR (qPCR) Assay for Measurement of BcenGI15 Excision Frequency

To determine the excision frequency of BcenGI15, qPCR assays were performed by using two sets of primer pairs. The primer pair exc_F/exc_R were used to quantify the copy number of excised BcenGI15 that will amplify the *attP* site and *rpoB*_F/*rpoB*_R were used to quantitate the total chromosome number in each genomic DNA sample, as this primer will amplify the single copy *rpoB* gene (Supplementary Table 1). Genomic DNA was isolated using ZR Fungal/Bacterial DNA MiniPrep Kit (Zymo Research Corporation, Orange, CA, USA) from *B. cenocepacia* IPCU-A by harvesting the cells at exponential growth phase (OD_600_ of 0.9) and quantified using Qubit 2.0 Fluorometer (Invitrogen, Carlsbad, CA, USA). qPCR was performed on Eppendorf Mastercycler ep realplex system (Eppendorf, Hamburg, Germany) using the Platinum SYBR Green qPCR SuperMix-UDG (Invitrogen, Thermo Fisher Scientific Inc., Waltham, MA, USA) in a 10 μl reaction. The cycling protocol was as follows: UDG incubation (50°C, 2 min), initial activation (95°C, 2 min) and 40 cycles of denaturation (95°C, 15 s) and combined annealing/extension (64°C, 60 s). The fluorescent data was collected at last step. The PCR products were subjected to the melt curve analysis to ensure no off targeted priming and specific amplification. The standard curve was prepared using 10-fold serial dilution of gDNA and was used to quantify the amount of target present in unknown. Standard curve preparation and calculation of quantity (copies) were done using Eppendorf Realplex software v.2.2. The excision frequency was determined as the copy number of the excised BcenGI15 divided by the total chromosome copy number.

### Nucleotide Sequence Accession Numbers

Whole genome sequences of seven isolates have been submitted to DDBJ/EMBL/GenBank under accession number JYMW00000000, JYMX00000000, JYMY00000000, JYMZ00000000, JYNA00000000, JYNB00000000, and JYNC00000000. Figshare link to download the assembled genomes is https://figshare.com/s/68ee97a7de63cff6e610.

## Results and Discussion

### Whole Genome Sequencing of Nosocomial Bcc Isolates

We sequenced and analyzed the whole genome of seven Bcc isolates from PICU, NICU and PW of the Topiwala National Medical College & BYL Nair Charitable Hospital, Mumbai. Five isolates were obtained from the blood cultures of different patients at different time intervals (BC-3, BC-19, BC-21, BC-40, BC-42) and two were obtained from swabs collected from the upper surface of the two-different amikacin vial rubber caps (IPCU-A and IPCU-B) during routine surveillance. Illumina reads were *de novo* assembled into high-quality draft genomes with coverage ranging from the 100× to 121×. WGS revealed that all the isolates of Bcc have GC content around 66% and genome size of around 8.6 Mb except one isolate BC-3 having the genome size of 7.8 Mb. General genomic features of nosocomial isolates of Bcc are mentioned in **Table 1**, and assembly statistics are mentioned in Supplementary Table 2.

**Table 1:** Genomic features of nosocomial *B. cenocepacia* isolates sequenced in the present study.

Isolate	BC-3	BC-19	BC-21	BC-40	BC-41	IPCU-A	IPCU-B
Genome size(bp)	789 051 3	866 638 9	865 987 9	866 833 2	868 550 9	866 941 6	866 729 9
GC (%)	67.13	66.41	66.41	66.41	66.37	66.41	66.41
CDS	7425	8075	8076	8096	8096	8088	8077
No. of rRNA	6	6	7	8	8	8	5
No. of tRNA	57	58	59	59	56	60	59
Isolation source	Blood culture	Blood culture	Blood culture	Blood culture	Blood culture	Amikacin vial rubber cap	Amikacin vial rubber cap
NCBI accession	JYMW00000000	JYMX00000000	JYMY00000000	JYMZ00000000	JYNA00000000	JYNB00000000	JYNC00000000

### Whole Genome-Based Species Identification of Bcc Isolates

Identifying species status of Bcc members is a challenging task because of their phenotypic similarities. To identify species status of nosocomial isolates of Bcc, we used ANI and dDDH, which are emerging as powerful genomic tools to assign species status. For species delineation, a cut-off of 94–96% and 70% for ANI and dDDH are used, respectively (Kim et al., 2014). A comparison of ANI of all seven isolates with type strain *B. cenocepacia* J2315^T^ showed the ANI values 98% with *B. cenocepacia* J2315^T^ and 90% with *B. cepacia* ATCC 25416^T^, which is next close species of *B. cenocepacia* and used as an outgroup (Supplementary Figure 1). Further, DDH values were >70% with *B*. *cenocepacia* J2315 and <70% with *B. cepacia* ATCC 25416^T^ (Supplementary Table 3). It suggested that all nosocomial isolates belonged to *B. cenocepacia*, as values were much above the cut-off for species delineation. Whole genome alignment of *B. cenocepacia* isolates with complete genome of *B. cenocepacia* J2315^T^ as the reference strain showed high-level synteny and increased genome size (except BC-3) (Supplementary Figure 2).

### SNP Analysis of Nosocomial Bcc Isolates

To understand the clonal nature of nosocomial isolates of *B. cenocepacia*, we analyzed the SNPs in core genome. SNPs distance comparison matrix obtained through CSI phylogeny 1.1a is mentioned in **Table 2**. Accordingly, four blood culture isolates differed by only two SNPs when compared with isolates from amikacin vial rubber cap (IPCU-A and IPCU-B). Differences in only a few SNPs suggested a common origin and clonality of these isolates indicating the epidemiological link of four blood isolates to amikacin vials’ rubber cap isolates. In contrast, more than 29000 SNPs observed in case of BC-3 suggested that this isolate is epidemiologically not related, and acquired by the patient from a different source.

**Table 2:** Single nucleotide polymorphism (SNP) distance matrix among seven nosocomial *B. cenocepacia* isolates calculated using CSI Phylogeny 1.1a web server.

	BC-19	BC-21	BC-40	BC41	IPCU-A	IPCU-B	BC-3
BC-19	0	1	0	2	2	2	29044
BC-21	1	0	1	1	1	1	29045
BC-40	0	1	0	2	2	2	29044
BC41	2	1	2	0	2	2	29046
IPCU-A	2	1	2	2	0	0	29046
IPCU-B	2	1	2	2	0	0	29046
BC-3	29044	29045	29044	29046	29046	29046	0

### Phylogenetic Relationship with Other *B. cenocepacia*

**FIGURE 1:**
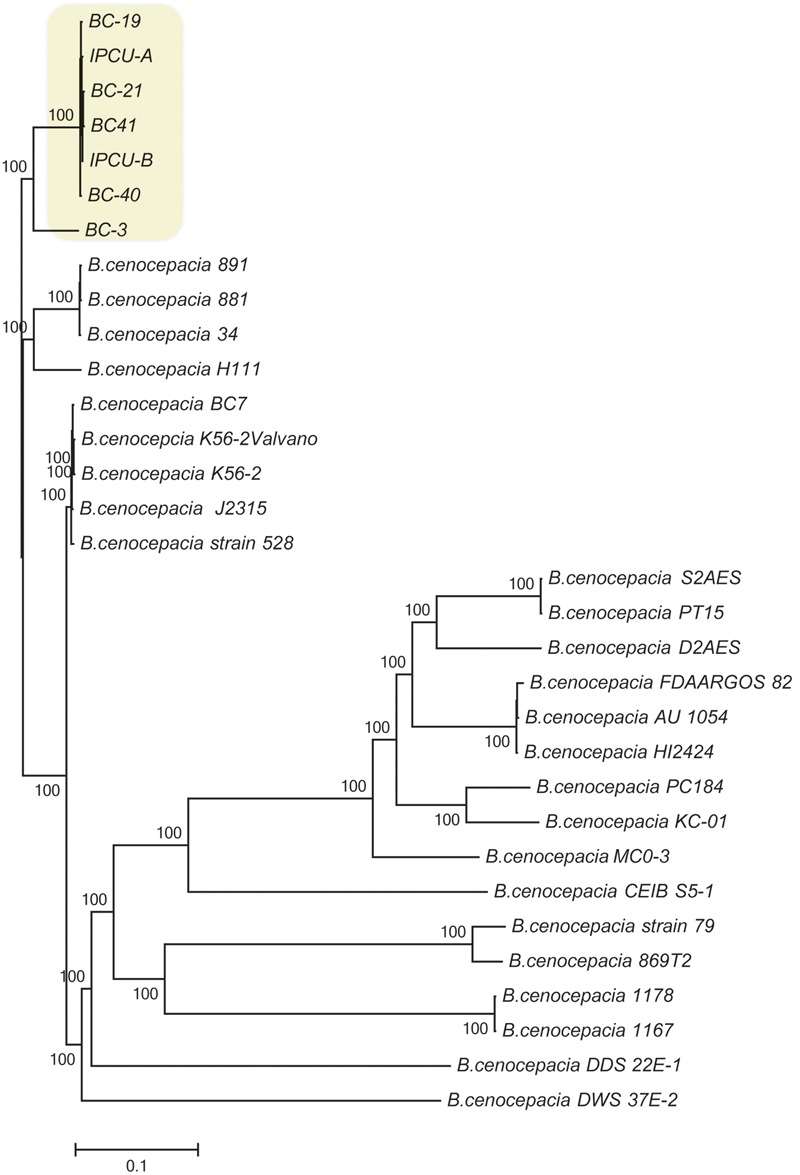
**Phylogenomic analysis of nosocomial *B. cenocepacia* isolates with other *B. cenocepacia*.** Phylogenetic tree based on SNPs in core genomes of nosocomial *B. cenocepacia* isolates and *B. cenocepacia* genome sequences from NCBI database was constructed. CSI Phylogeny web server was used to perform SNP detection and phylogenetic inference. Branch lengths correspond to numbers of nucleotide substitutions per site. A phylogenetically distinct cluster of nosocomial isolates of *B. cenocepacia* from this study is highlighted in the yellow background.

To further characterize the isolates of this study, SNP-based phylogenetic analysis was carried out by including 25 publically available genome sequences of *B. cenocepacia* isolates from diverse geographical locations (Supplementary Table 4). SNPs in core genome were identified using complete genome of *B. cenocepacia* J2315^T^ as a reference strain. Phylogenetic tree based on SNPs showed that four isolates from clinical cases (BC-19, BC-21, BC-40, BC-41) and two isolates from amikacin vial rubber cap (IPCU-A and IPCU-B) belonged to one phylogenetic cluster, which was distinct from other global isolates from NCBI database (**Figure 1**). Even the variant BC-3 isolate was also phylogenetically distinct from global isolates but much closer to clonal isolates from this study. This indicated that nosocomial isolates of *B. cenocepacia* obtained in the present study were phylogenetically distinct.

### Comparative Genomics Based Identification of a Novel Genomic Island (BcenGI15) in Nosocomial Isolates of *B. cenocepacia*

**FIGURE 2:**
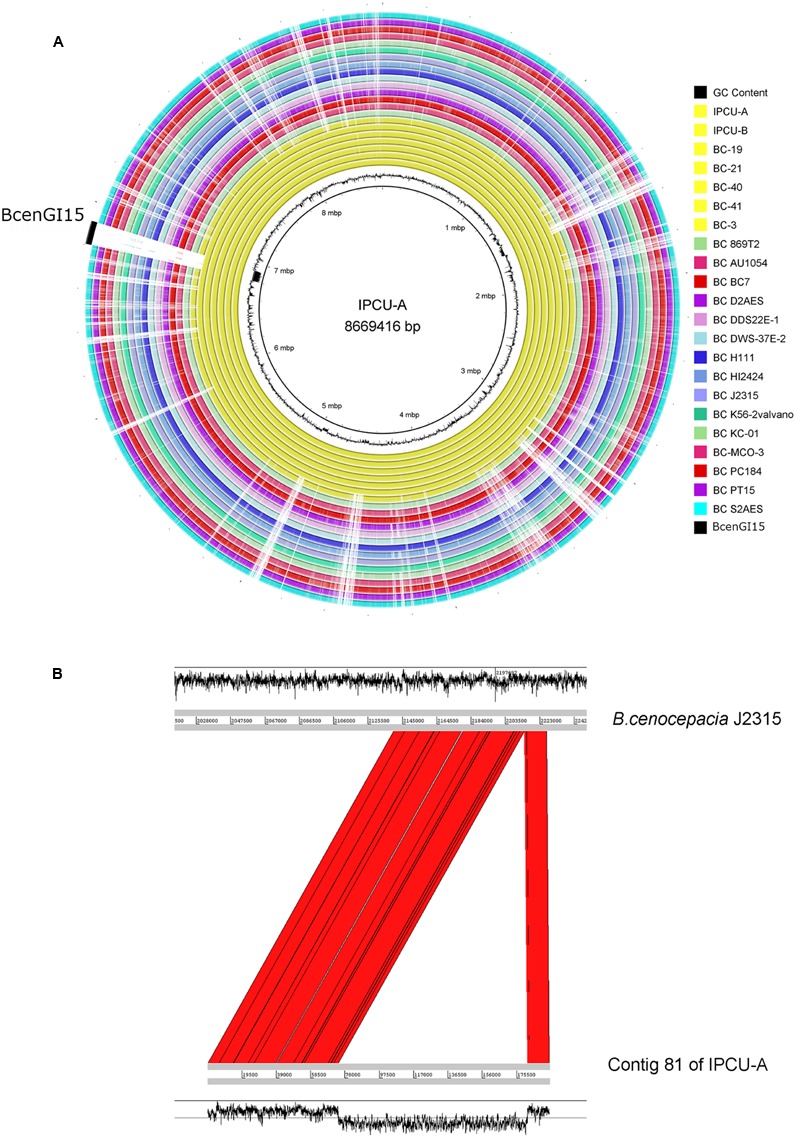
**Circular representation of *Burkholderia cenocepacia* genomes to mark the genomic location of BcenGI15. (A)** Genomes of various *B. cenocepacia* (BC) strains are visualized in the circular form by mapping on strain IPCU-A as a reference. One ring represents each genome; yellow rings represent the nosocomial isolates sequenced in this study, and all other rings are of the genomes of other *B. cenocepacia* (BC) strains available at NCBI database. The GC content (%) of IPCU-A is shown by a ragged inner circle in black. The novel genomic island (BcenGI15) associated with nosocomial isolates is compared across all the genomes. **(B)** Artemis comparison of contig 81 of IPCU-A containing BcenGI15 with reference genome *B. cenocepacia* J2315. Red color shows the region of forward matches. The GC content (%) graph is drawn in black to show the variability.

The genomes of nosocomial isolates of Bcc were compared with publicly available genomes from NCBI database (Supplementary Table 4). The comparison revealed the presence of a large genomic region, which is exclusively present in unique clonal lineage identified in the present study (**Figure 2A**). Overall GC content of identified unique region is 45%, which is dramatically lower than genomic GC content (67%) of *B. cenocepacia.* It suggests that this region has genes that are not native and have been acquired by HGT. Interestingly, this unique genomic region is integrated into tRNA^Leu^ (TAG) in the vicinity of phytoene synthase gene and as a result of this integration it is flanked by 9 bp direct repeats (5′-GGTTTAGGT-3′) representing *attL* and *attR* sites. However, there is one copy of tRNA^Leu^, which is an integral part of this unique genomic region. This unique genomic region is 107277 bp in size and inserted at position 12559–119836 of contig 81 of isolate IPCU-A. Comparison of the contig 81 of IPCU-A with complete genome of *B. cenocepacia* J2315, which lacks this region, revealed that unique region is an internal part of a chromosome and has similar flanking genomic regions (**Figure 2B**). This region is identical in all clonal nosocomial isolates under this study and shows no structural variations among them (Supplementary Figure 3).

**FIGURE 3:**
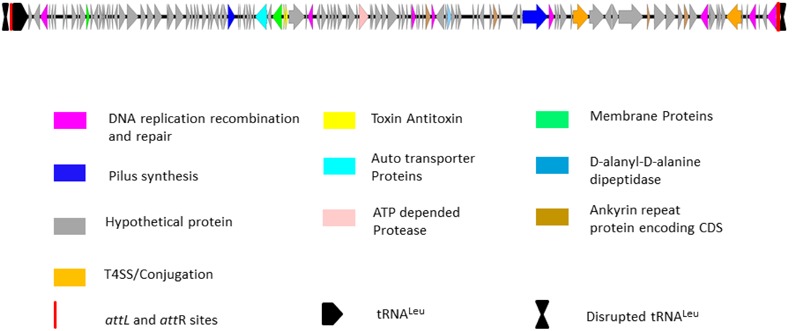
**Schematic organization of genomic island BcenGI15.** Genetic organization of BcenGI15 of IPCU-A is integrated at 107278-119836 (Contig81). The *attL* and *attR* sites generated after insertion at tRNA^Leu^ are represented as red rectangles. ORFs with different functions are colored differently. ORFs are represented by arrows drawn approximately to scale.

The unique genomic region of isolate IPCU-A contains 125 open reading frames, amongst which 106 are hypothetical. Further manual annotation of ORFs by using BLASTP suggests that ORFs with assigned functions are involved in DNA integration, excision, stabilization, conjugational DNA processing and regulation (Supplementary Table 5). Schematic organization of predicted ORFs of this unique genomic region is shown in **Figure 3**. This unique genomic region identified through comparative genomics shows characteristic features of a GEI; a large genomic segment with altered GC content compared to the genome, insertion at tRNA, flanking direct repeats and genes for genetic mobility like integrase and conjugation associated proteins. These features suggest that this unique genomic region is GEI. Fourteen GEIs, namely BcenGI1 to BcenGI14, were identified in the reference genome *B. cenocepacia* J2315 (Holden et al., 2009), thus in continuation we named this novel GEI as BcenGI15, and it has been further referred as “BcenGI15.”

Chromosomal integration and excision of GEI is usually mediated by a site-specific recombinase/integrase, which is a tyrosine recombinase in most cases, while in some cases it can also be a serine recombinase or DDE transposases. BcenGI15 also harbors an integrase gene at one extremity. An alignment of protein sequences of integrase with characterized tyrosine recombinase confirmed that it belongs to tyrosine recombinase superfamily. It has a characteristic RHRH tetrad with conserved nucleophilic tyrosine at C-terminus, a characteristic feature of this protein superfamily (Esposito and Scocca, 1997) (Supplementary Figure 4). Phylogenetic analysis with tyrosine recombinase from GenBank database revealed it to be a novel tyrosine recombinase as it is phylogenetically distinct and diverse from recombinase of bacteriophage, integrons and ICEs (Supplementary Figure 5).

Transfer of GEI between bacteria is usually mediated by conjugation and in some cases by transduction. BcenGI15 also harbors orthologs of genes involved in the conjugational DNA processing and transfer like *tra*A (relaxase), *traD, TraG*, and *traY*. Conjugational DNA transfer is mediated by the Type IV secretory system encoded by multiple genes on single operon. Only a few genes involved in conjugation are present on BcenGI15, suggesting that it lacks complete machinery required for the conjugational transfer of this GEI. Vertical transmission and maintenance of excised GEI is mediated by toxin–antitoxin system (Hayes, 2003). One such type II toxin–antitoxin system is present in BcenGI15, which encodes toxin and antitoxin of ParE and YefM family, respectively. Interestingly, BcenGI15 encode five ORFs that code for putative methyltransferase (d*pnA*), which is known to play a crucial role during transfer of GEI when foreign DNA is unmethylated (Johnston et al., 2013), and it also provides resistance against bacteriophages (Balado et al., 2013). Like methyltransferase, there is another gene for anti-restriction protein (ArdA), which is necessary for prevention of cleavage by restriction enzymes (Belogurov et al., 2000).

### BcenGI15 Harbor Putative Pathogenicity-Related Genes

BLASTP analysis of ORFs in BcenGI15 revealed that many of them are orthologs of characterized genes involved in pathogenesis-related adaptations. (Supplementary Table 5). BcenGI15 encodes a gene for auto transporter adhesion having YadA domain known to be important in the pathogenesis related functions like adherence, biofilm formation, invasion, survival within eukaryotic cells, serum resistance, and cytotoxicity (Mil-Homens and Fialho, 2011; Mil-Homens et al., 2014). Four genes of BcenGI15 encode ankyrin repeat proteins (CD0024) that are abundant in eukaryotes and play a role in protein–protein interactions (Li et al., 2006). In bacteria, these are major proteins secreted by Type IV secretory systems and involved in modulation of host cell functions (Nakayama et al., 2008). This GEI has one gene that encodes for a protein having a conserved domain for SGNH hydrolase, which belongs to a diverse family of lipases and esterases. In *Pseudomonas aeruginosa*, SGNH hydrolases are involved in acetylation of alginate, which is a long sugar polymer and the main component of *Pseudomonas* biofilm (Baker et al., 2014). Acetylation of alginate during its biosynthesis makes bacterial biofilm less susceptible to antibiotics, disinfectants and enable them to evade the immune system of a host (Ertesvåg, 2015). GEI also harbors gene *vanY* that encodes for D-alanyl-D-alanine carboxypeptidase (COG 2173) of VanY superfamily known to be involved in the cell wall biogenesis and resistance to glycopeptide antibiotic vancomycin. In *Brucella* spp., D-alanyl-D-alanine carboxypeptidase contributes to its intracellular replication and resistance against nitric oxide (Kikuchi et al., 2006).

These observations for the presence of putative pathogenesis related genes on this GEI indicate a putative role of this GEI in pathogenicity. Further mutation and virulence studies in an experimental animal model are required to demonstrate the role of BcenGI15 in pathoadaptation of *B. cenocepacia*.

### BcenGI15 Actively Excising from Genome as Extrachromosomal Circular Form

**FIGURE 4:**
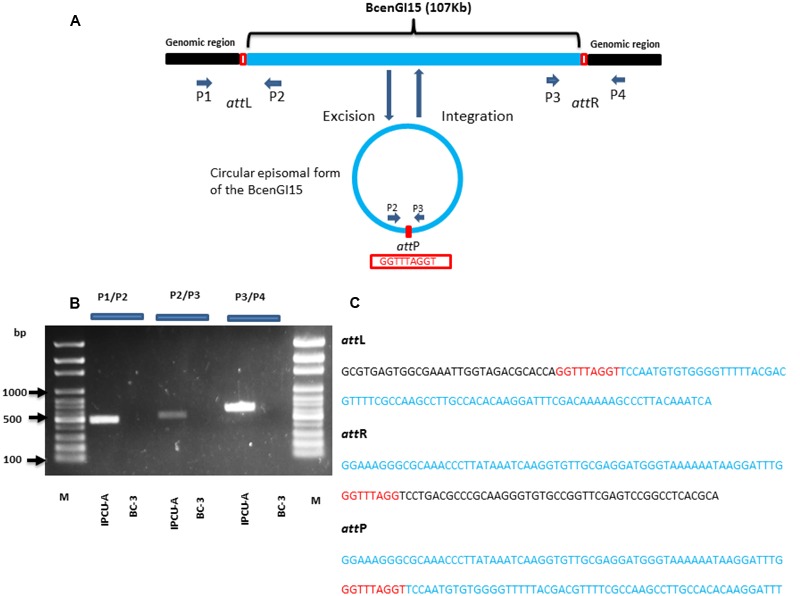
**Schematic representation of the integration and excision of BcenGI15. (A)** Schematic representation of BcenGI15 integrated into the genome (top) and excised (bottom) form. Chromosomal part is shown as a black and BcenGI15 part is in solid blue lines. The red box shows the *attL* and *attR* sites along with *attP* site in excised form. Primers used in PCR assay are shown with small arrowheads. ORFs are represented by arrows drawn approximately to scale. **(B)** 1% Agarose gel showing PCR products obtained from primer pair P1/P2, P2/P3, P3/P4 covering *attL, attP*, and *attR* sites, respectively. Lanes: M, 100 bp ladder; 1, *attL* site of IPCU-A; 2, *attL* site of BC-3 (No amplification); 3, circular episomal form of BcenGI15 of IPCU-A; 4, circular episomal form of BcenGI15 BC-3 (No amplification); 5, *attR* site of IPCU-A;6, *attR* site of BC-3 (No amplification). **(C)** The nucleotide sequence of PCR products containing *attL, attR*, and *attP* sites. DNA sequence belonging to the genomic island (BcenGI15), repeats of *att* sites and core chromosomal DNA are shown in blue, red and black color, respectively.

To investigate whether BcenGI15 has the ability to excise from the genome as circular extrachromosomal or episomal excision product, we used nested PCR approach. Primers P2 and P3 were designed in such way that they were oriented toward the left, and a right junction of BcenGI15 (**Figure 4A**), which produced amplicons only in the circular excised form of BcenGI15. These primers amplified a product of 512 bp only if BcenGI15 got excised from the chromosome and got circularized by recombination between *attL* and *attR* sites. A PCR fragment of the desired size was obtained from IPCU-A, but not from isolate BC-3 (**Figure 4B**). By using two different sets of primers P1/P2 (covering *attL*) and P3/P4 (covering *attR*) for PCR amplification, we confirmed the insertion of BcenGI15 in the genome (**Figures 4A,B**). The presence of *attL, attR* and *attP* sites in amplified PCR products was confirmed by sequencing (**Figure 4C** and Supplementary Figure 6). This molecular evidence reveals that BcenGI15 is capable of excising from the genome and forming extrachromosomal circular form. To further determine the excision frequency, we used qPCR assays. The copy number of the excised BcenGI15 and total number of chromosomes was calculated by using primer pairs that will amplify the *attP* site and *rpoB*, respectively (Supplementary Table 1). The relative ratio (excision frequency) of the number of excised circular BcenGI15 to that of total number of chromosomes for *B. cenocepacia* IPCU-A cells grown in LB medium was 8.6 × 10^-7^.

Thus, BcenGI15 has the ability of actively excising from the genome and forming episomal circular form suggesting its capability of mobilizing to other strains. Although it lacks complete machinery required for conjugational transfer, it may depend on another helper conjugative plasmid for conjugation. Further experimentation focussing on conjugational transfer of BcenGI15 is required to conclude this GEI as ICE or IME.

### *Burkholderia pseudomallei* Strain EY1 Harbors Homolog of BcenGI15

**FIGURE 5:**
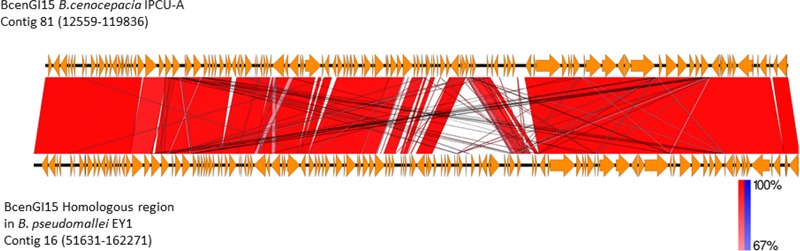
**Comparison of the genetic organization of BcenGI15 with its homolog in *Burkholderia pseudomallei* strain EY1.** Comparison of genetic organization BcenGI15 (top panel) and its homolog in the genome of *Burkholderia pseudomallei* strain EY1 (bottom panel). Arrows represent predicted CDS. Highly conserved regions determined by pairwise BLASTN comparisons with *E*-values lower than 0.001 were plotted. Regions with forward and reverse matches are indicated by red and blue shades, respectively, with color intensity indicating nucleotide identity levels (from 67 to 100%). The absence of red and blue area denotes no homology.

BLASTP analysis of the recombinase/integrase of GEI against a non-redundant database revealed that this tyrosine recombinase of GEI is highly identical (BLASTP, cover 100%, identity 99%, *E*-value 0.0) with recombinase from *B. pseudomallei* strain EY1. Analysis of the corresponding genomic region in *B. pseudomallei* strain EY1 (GenBank accession number: GCA_001212405.1) revealed that this region is similar to BcenGI15, which is also integrated at tRNA^Leu^ (TAG) in the vicinity of phytoene synthase gene and flanked by 9 bp direct repeat (5′-GGTTTAGGT-3′). This homologous GEI of *B. pseudomallei* strain EY1 is 110640 bp in size and inserted at position 51631–162271 of contig 16. *B. pseudomallei* strain EY1 was isolated from melioidosis diseased patient’s blood sample in 1997 from Malaysia (Nandi et al., 2014). Alignment of BcenGI15 with its homologs region in *B. pseudomallei* strain EY1 showed a high level of synteny (**Figure 5**). The overall identity of BcenGI15 with its homolog in *B. pseudomallei* strain EY1 is 83%, which suggests its acquisition from a common source or there is possibility of interspecies movement between these strains. Interestingly, despite the availability of a large number of genomes of *B. cenocepacia* and *B. pseudomallei*, presence of this GEI is unique to *B. pseudomallei* strain EY1 and phylogenetically distinct *B. cenocepacia* isolates from the present study.

## Conclusion

Genome-based comprehensive analysis of nosocomial isolates of Bcc from a hospital in India revealed a clone that is phylogenetically distinct from other *B. cenocepacia* isolates whose genome sequences are available in public domain. Further comparative genomics revealed the presence of a novel GEI BcenGI15 harboring putative pathogenesis-related genes. BcenGI15 is capable of excising from the genome and forming circular intermediate form suggesting it to be a mobile GEI. The presence of GEI similar to BcenGI15 in a *Burkholderia pseudomallei* strain EY1 suggested the interspecies movement of GEI between variant isolates of pathogenic species of genus *Burkholderia*. This study will aid in understanding the growing concern of nosocomial infections due to Bcc by accelerating our knowledge of epidemiology and the evolution of pathogenic species belonging to the genus *Burkholderia.*

## Author Contributions

SMa, LD, and JS carried out isolation and clinical investigation of nosocomial isolates. SK and LS performed initial identification and molecular typing. PPP and SMi performed whole genome sequencing and bioinformatics analysis. PPP drafted the manuscript with inputs from SMa, SMi, VG, and PBP. SMa, VG, and PBP conceived, designed and coordinated study. All authors contributed, read and approved the final manuscript.

## Conflict of Interest Statement

The authors declare that the research was conducted in the absence of any commercial or financial relationships that could be construed as a potential conflict of interest.

## Footnotes

1 http://ggdc.dsmz.de/distcalc2.php

2 https://software.broadinstitute.org/GENE-E/

3 https://cge.cbs.dtu.dk/services/CSIPhylogeny/

4 http://www.genomicepidemiology.org/

5 http://rast.nmpdr.org/rast.cgi
