# Supplementary material for: Genomics Reveals a Unique Clone of Burkholderia cenocepacia Harboring an Actively Excising Novel Genomic Island
Source: Front Microbiol. 2017 Apr 6;8:590. doi: 10.3389/fmicb.2017.00590 (PMC5382208; doi:10.3389/fmicb.2017.00590)
Supplement: Supplementary file 1 [file Data_Sheet_1.docx]

**Supplementary Information**

**Genomics reveals a unique clone of *Burkholderia cenocepacia* harbouring an actively excising novel genomic island**

Prashant P. Patil^1^, Swapna Mali^2^, Samriti Midha^1^, Vikas Gautam^3*^, Lona Dash^2^, Sunil Kumar^3^, Jayanthi Shastri^2^, Lipika Singhal^3$^, Prabhu B. Patil^1*^

^1^ Laboratory of Bacterial Genomics and Evolution, CSIR-Institute of Microbial Technology, Chandigarh, India.

^2^Department of Microbiology, Topiwala National Medical College & B. Y. L. Nair Charitable Hospital, Mumbai, India.

^3^Post Graduate Institute of Medical Education and Research, Chandigarh, India.

^$^Current affiliation: Department of Microbiology, Government Medical College & Hospital, Chandigarh.

Correspondents: [pbpatil@imtech.res.in](mailto:pbpatil@imtech.res.in) & [r_vg@yahoo.co.uk](mailto:r_vg@yahoo.co.uk)

No of Supplementary Figures: 6

No of Supplementary Tables: 5

**Supplementary Figure 1**

**
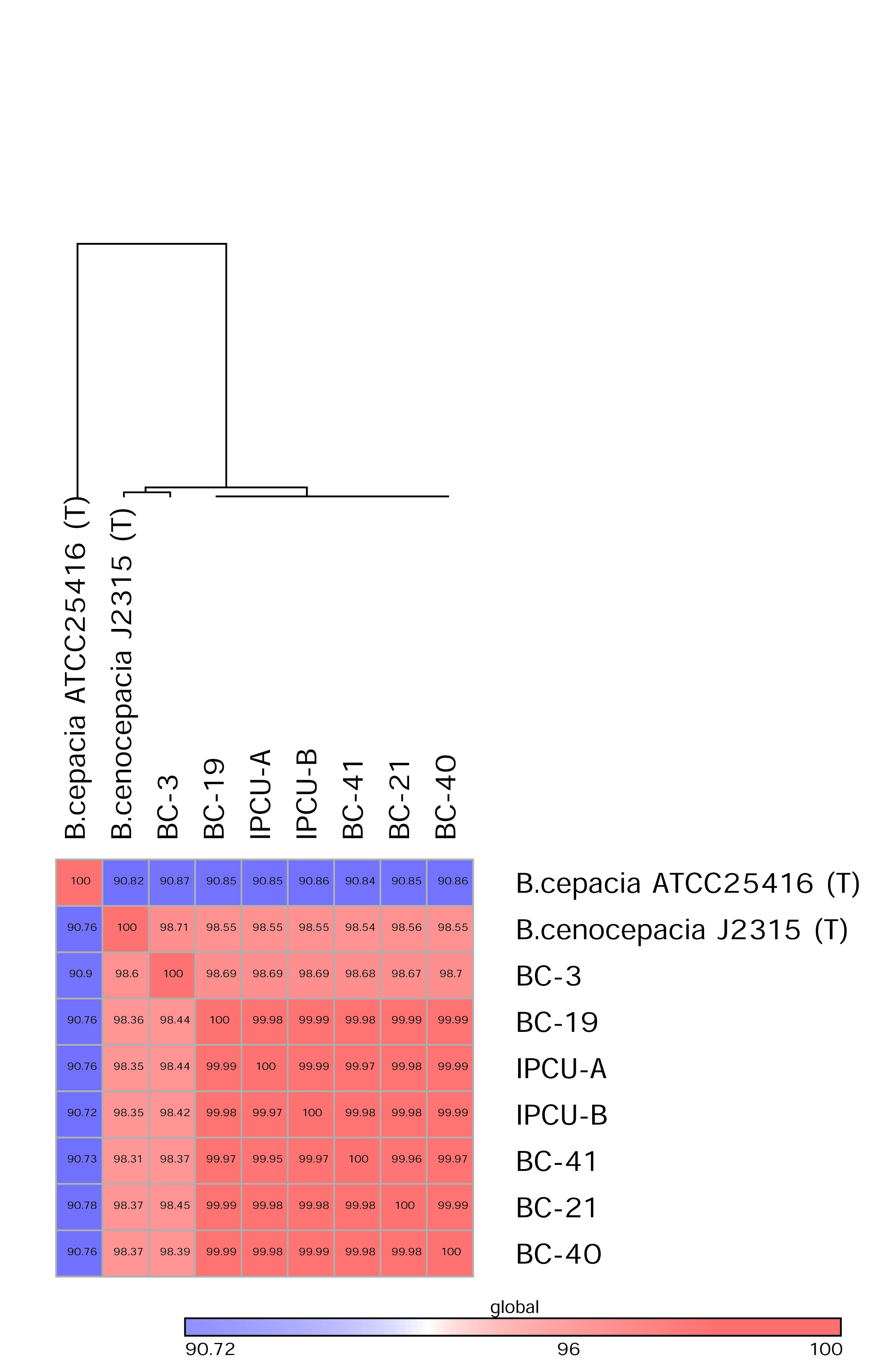
**

**Supplementary Figure 1: Genome-based assessment of species of nosocomial Bcc isolates.**

Heat map of Average Nucleotide Identity (ANI) values of nosocomial isolates with *Burkholderia cenocepacia* J2315^T^ and *Burkholderia cepacia* ATCC25416^T^*.*

**Supplementary Figure 2**

**
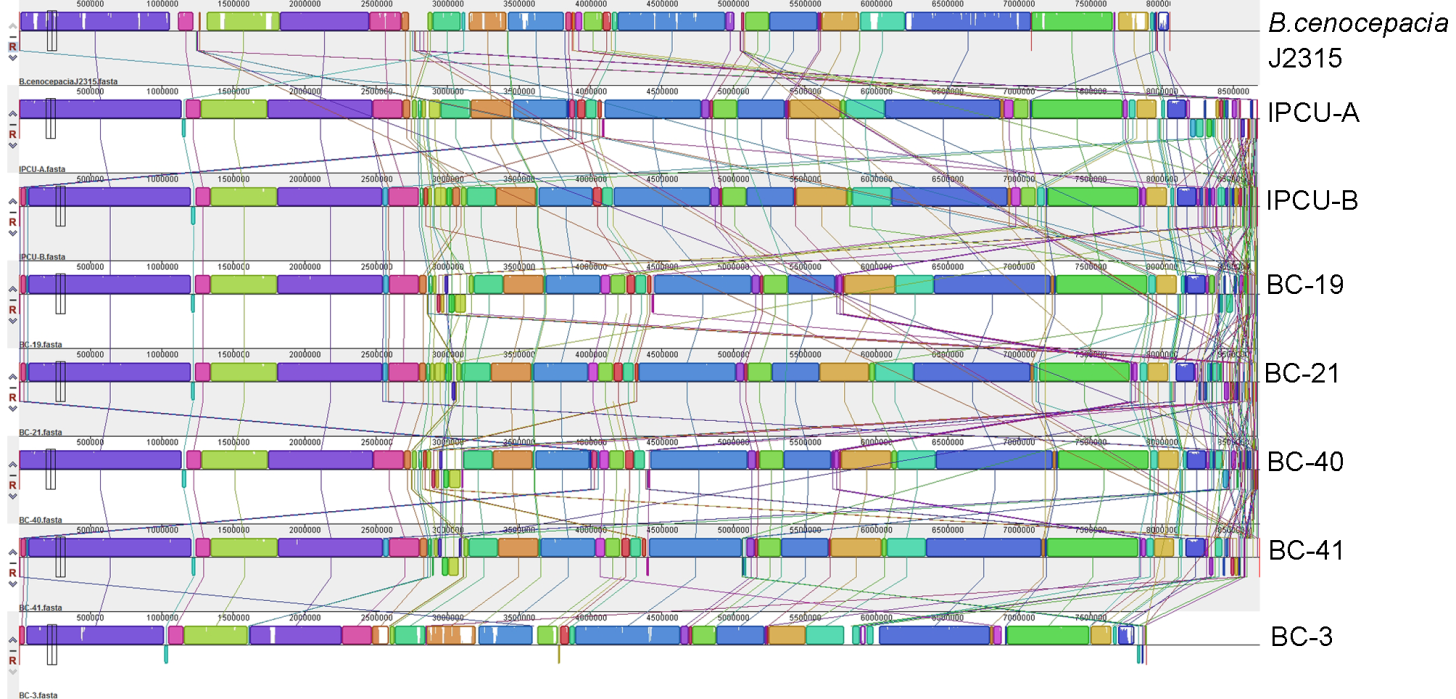
**

**Supplementary Figure 2: Multiple genome alignment of nosocomial isolates of *B. cenocepacia* with reference genome *Burkholderia cenocepacia* J2315 by using progressive Mauve**

Rectangles of same colour represents local collinear blocks (LCBs) of conserved sequences amongst strains. Connecting lines between different genomes represent genomic rearrangements. Alignments of LCBs above or below the basaeline depicts forward and reverse orientations of LCBs. The height of colour graphs in LCBs represents percent identity and absence of colour or white region shows strain-specific regions. Sequences which are not placed within an LCB are unique to a particular strain.

**Supplementary Figure 3:**

**
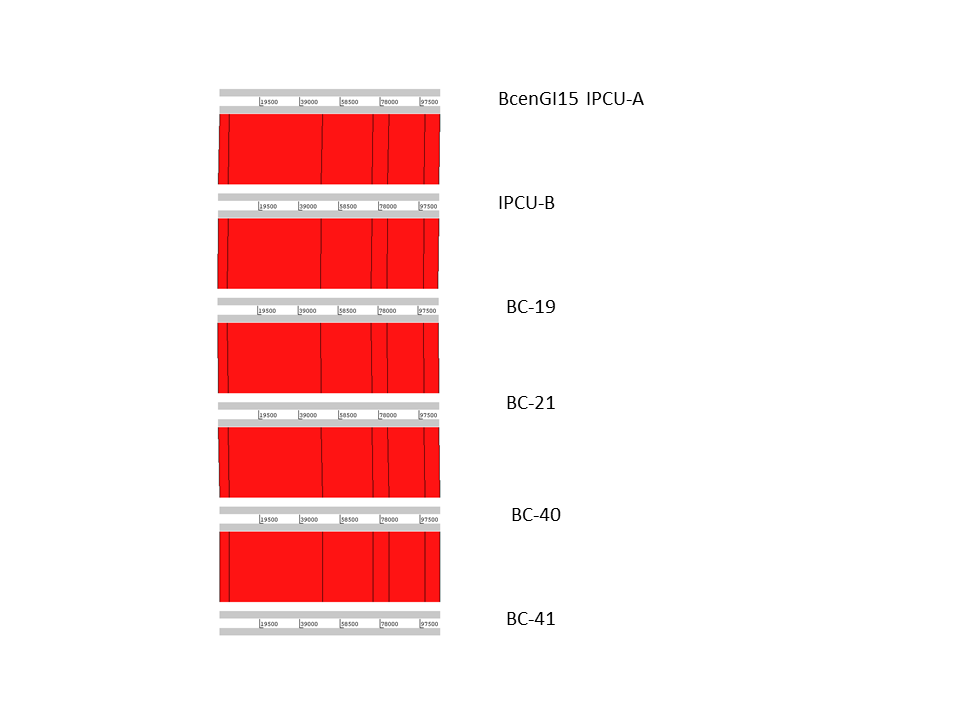
**

**Supplementary Figure 3:** Artemis comparison of the unique genomic region from clonal nosocomial isolates of *B. cenocepacia*. Red colour shows region of forward matches.

**Supplementary Figure 4:**

**
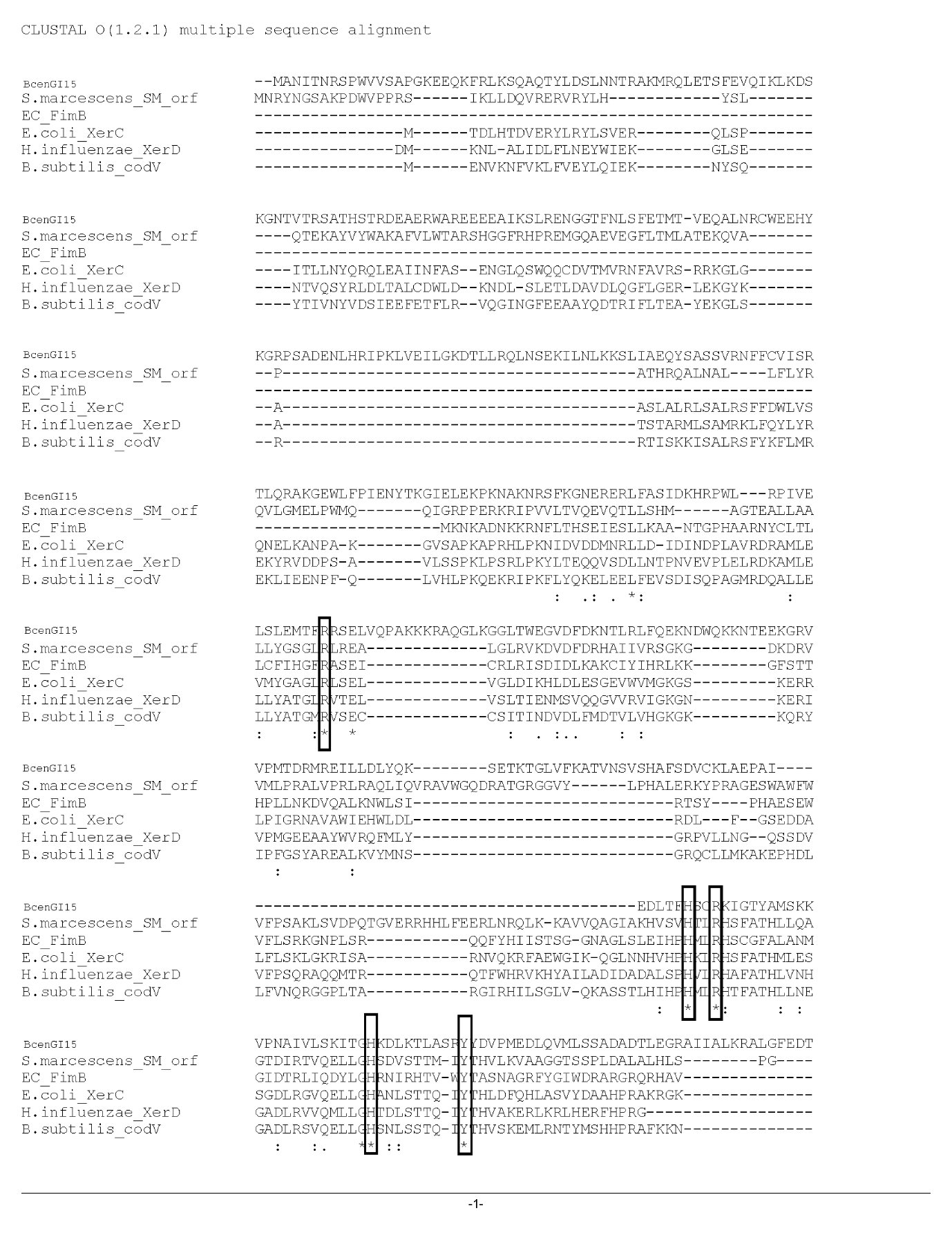
**

**
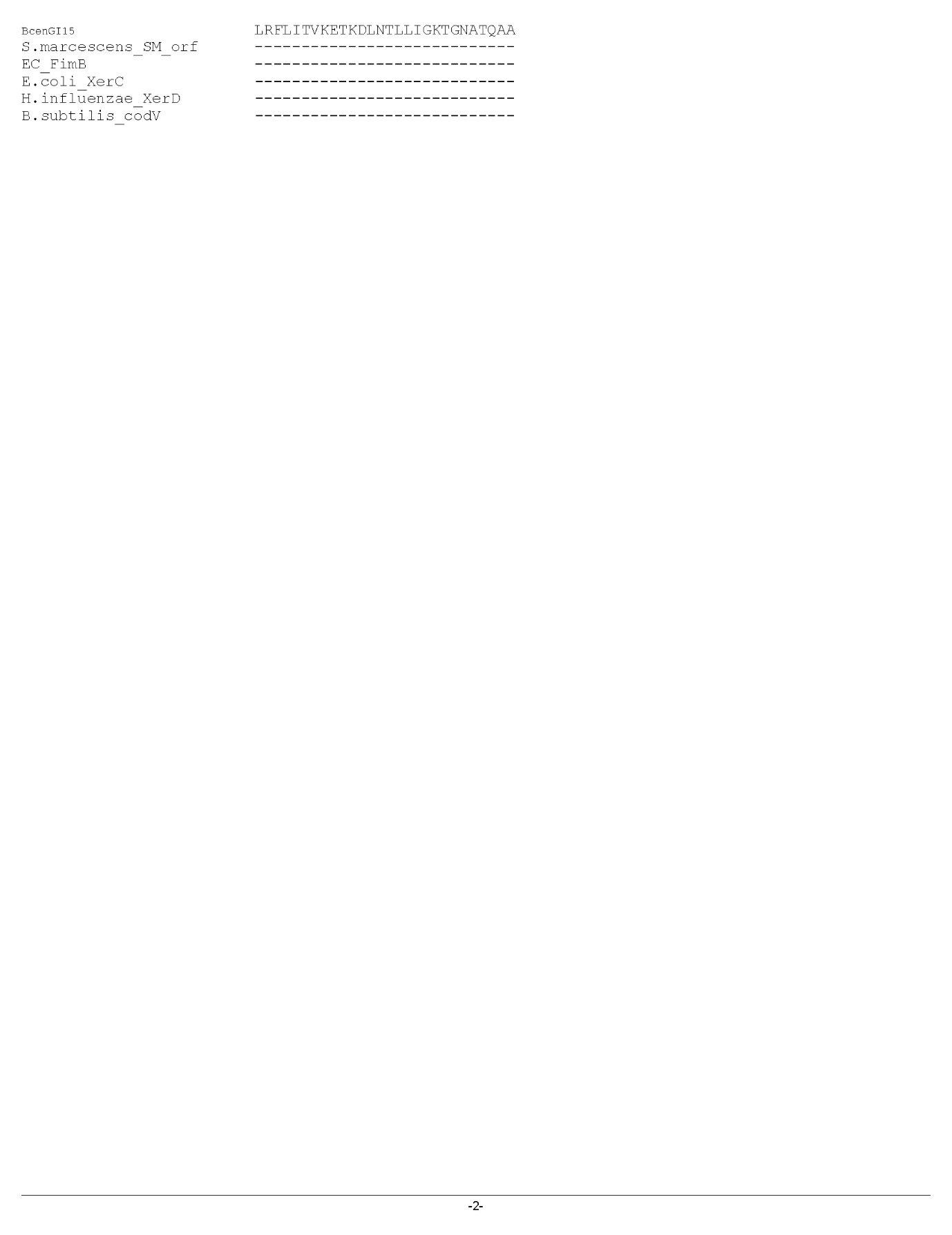
Supplementary Figure 4**: Alignment of protein sequences of recombinase of BcenGI15 with characterized tyrosine recombinase family illustrating conserved RHRH tetrad and conserved tyrosine which is characteristic of tyrosine recombinase superfamily**.**

**Supplementary Figure 5:**

**
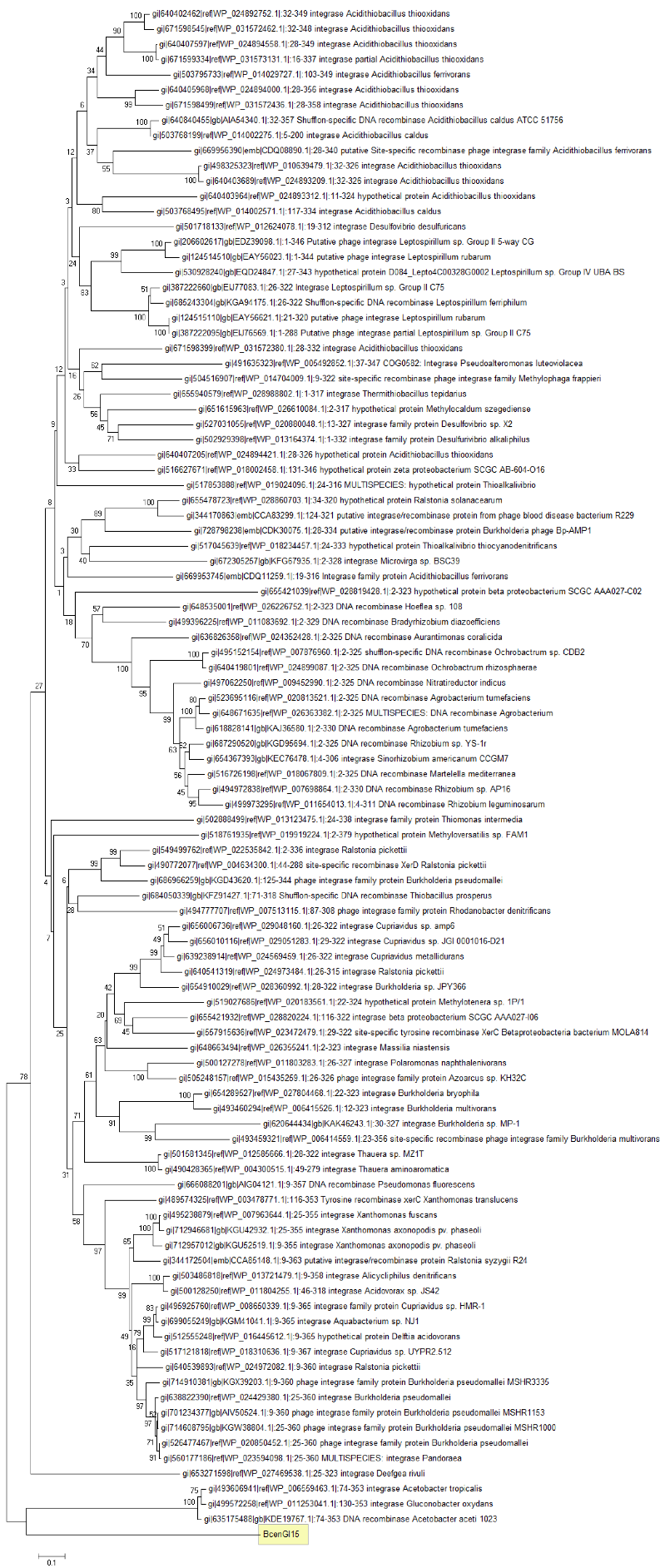
**

**Supplementary Figure 5:** Phylogenetic tree of tyrosine recombinase like protein from phages, prophages and ICE available at GeneBank database with recombinase of BcenGI15 (highlighted). The scale bar represents a 0.1 substitution per nucleotide position.

**Supplementary Figure 6:**

**A) *attL* (Primer pair P1/P2)**

**
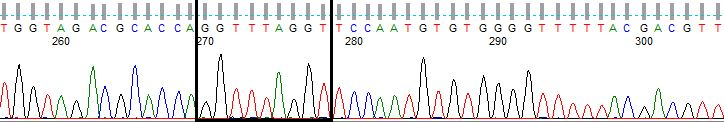
**

**B) *attR* (Primer Pair P3/P4)**


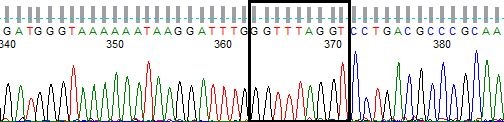


**C) *attP* (Primer Pair P2/P3)**

**
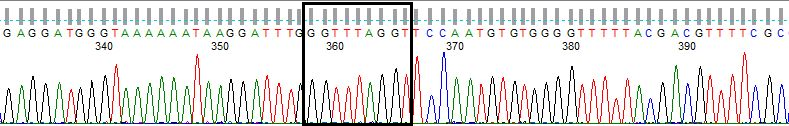
**

**Supplementary Figure 6:** Sanger Sequencing chromatograms of PCR products containing *attL*, *attR* and *attP* sites (boxed).

**Supplementary Table 1**

List of primers used in this study to detect integration, circular extrachromosomal form and to calculate the excision frequency of BcenGI15.

| **Detection site** | **Primers** | **Sequence**  **(5’to 3’)** | **Genomic coordinates in IPCU-A** | **Amplicon (bp)** | **Annealing temperature** |
| --- | --- | --- | --- | --- | --- |
| *attL* | P1 | gccttttgctgacagtagtcg | 12253-12273 (Contig 81) | 476 | 60^0^C |
|  | P2 | CCAAACTCTCCCCAAACTGA | 12709-12728 (Contig 81) |  |  |
| *attR* | P3 | TGACAGTGTTTCCCTTGCT | 119486-119502 (Contig 81) | 637 | 60^0^C |
|  | P4 | AGAACGGAGATCGGAATGC | 1200702-120684 (Contig 81) |  |  |
| *attP* | P2 | CCAAACTCTCCCCAAACTGA | 12709-12728 (Contig 81) | 539 | 60^0^C |
|  | P3 | TGACAGTGTTTCCCTTGCT | 119486-119502 (Contig 81) |  |  |
| *attP* | exc_F | aaggcttggcgaaaacgtc | 12580-12598 (Contig 81) | 135 | 64^0^C |
|  | exc_R | ggagtttgggaaaaagtttgg | 119731-119751(Contig 81) |  |  |
| *rpoB* | *rpoB*_F | cgttcaccaagttccgttct | 13967-13986 (Contig 36) | 130 | 64^0^C |
|  | *rpoB*_R | aatgggaaataccgacgtga | 13857-13876 (Contig 36) |  |  |

**Supplementary Table 2**

Genome sequencing and assembly statistics data of nosocomial isolates of *B.cenocepacia*.

| **Isolate** | **Average Read Length (bp)** | **Total base pair in Reads** | **Total base pair in contigs** | **Coverage**  **(Fold)** | **Contig count** | **N50 (bp)** | **NCBI Accession** |
| --- | --- | --- | --- | --- | --- | --- | --- |
| IPCU-A | 218.81 | 959 276 120 | 866 941 6 | 110.65 | 192 | 104 499 | JYNB00000000 |
| IPCU-B | 224.05 | 883 599 973 | 866 729 9 | 101.95 | 183 | 117 034 | JYNC00000000 |
| BC-3 | 229.71 | 102 682 5 455 | 789 051 3 | 130.13 | 133 | 129 174 | JYMW00000000 |
| BC-19 | 233.75 | 865 791 174 | 866 638 9 | 99.90 | 177 | 112 864 | JYMX00000000 |
| BC-21 | 215.03 | 103 939 559 2 | 865 987 9 | 120.02 | 196 | 117 325 | JYMY00000000 |
| BC-40 | 225.64 | 105 713 302 6 | 866 833 2 | 121.95 | 187 | 120 623 | JYMZ00000000 |
| BC-41 | 254.44 | 108 583 122 8 | 870 928 7 | 125 | 280 | 110 592 | JYNA00000000 |

**Supplementary Table 3**

Digital DNA-DNA Hybridization (dDDH) values of nosocomial isolates with type strains *Burkholderia cenocepacia* J2315^T^ and *Burkholderia cepacia* ATCC 25416^T^

|  | **BC-3** | **BC-19** | **BC-21** | **BC-40** | **BC-41** | **IPCU-A** | **IPCU-B** | ***B.cenocepacia* J2315 ^T^** | ***B.cepacia* ATCC 25416 ^T^** |
| --- | --- | --- | --- | --- | --- | --- | --- | --- | --- |
| **BC-3** |  | 82.6 | 82.3 | 82.6 | 81.3 | 82.5 | 82.4 | 84.3 | 62.2 |
| **BC-19** |  |  | 100 | 100 | 100 | 100 | 100 | 76.8 | 55.3 |
| **BC-21** |  |  |  | 100 | 100 | 100 | 100 | 76.5 | 55.1 |
| **BC-40** |  |  |  |  | 100 | 100 | 100 | 76.8 | 55.3 |
| **BC-41** |  |  |  |  |  | 100 | 100 | 75.4 | 54.4 |
| **IPCU-A** |  |  |  |  |  |  | 100 | 76.7 | 55.3 |
| **IPCU-B** |  |  |  |  |  |  |  | 76.6 | 55.2 |
| ***B.cenocepacia* J2315^T^** |  |  |  |  |  |  |  |  | 59.0 |

**Supplementary Table 4**

The list of genomes of *Burkholderia cenocepacia* used in the global phylogenetic analysis along with NCBI nucleotide accession numbers.

| **Sr. No** | **Strain** | **Nucleotide Accession No** |
| --- | --- | --- |
| 1. | *Burkholderia cenocepacia J2315* (T*)* | NC_011001,NC_011000&NC_011002 |
| 2. | *Burkholderia cenocepacia HI2424* | NC_008543,NC_008542&NC_008544 |
| 3. | *Burkholderia cenocepacia BC7* | NZ_ALIZ00000000 |
| 4. | *Burkholderia cenocepacia AU 1054* | NC_008062,NC_008061&NC_008060 |
| 5. | *Burkholderia cenocepacia H111* | NZ_CAFQ00000000 |
| 6. | *Burkholderia cenocepacia PC184* | NZ_AAKX00000000 |
| 7. | *Burkholderia cenocepacia MC0-3* | NC_010515,NC_010512& NC_010508 |
| 8. | *Burkholderia cenocepacia K56-2Valvano* | NZ_ALJA00000000 |
| 9. | *Burkholderia cenocepacia* DDS-22E-1 | NZ_CP007783.1, NZ_CP007784.1 & NZ_CP007782.1 |
| 10. | *Burkholderia cenocepacia* DWS-37E-2 | NZ_CP007781.1, NZ_CP007780.1 & NZ_CP007779.1 |
| 11. | *Burkholderia cenocepacia* KC-01 | NZ_AWOP00000000 |
| 12. | *Burkholderia cenocepacia* PT 15 | NZ_AXZX00000000 |
| 13. | *Burkholderia cenocepacia* SA2ES | NZ_AXZY00000000 |
| 14. | *Burkholderia cenocepacia* D2AES | NZ_AXZZ00000000 |
| 15. | *Burkholderia cenocepacia* 869T2 | NZ_JJOA00000000 |
| 16. | *Burkholderia cenocepacia* CEIB 5s-1 | NZ_JTLT00000000 |
| 17. | *Burkholderia cenocepacia* K56-2 | NZ_LAUA00000000 |
| 18 | *Burkholderia cenocepacia* 1178_BCEN | NZ_JWAD00000000 |
| 19 | *Burkholderia cenocepacia* 1167_BCEN | NZ_JWAO00000000 |
| 20 | *Burkholderia cenocepacia* 34_BCEN | NZ_JVKX00000000 |
| 21 | *Burkholderia cenocepacia* 79_BCEN | NZ_JUSV00000000 |
| 22 | *Burkholderia cenocepacia* 881_BCEN | NZ_JUPN00000000 |
| 23 | *Burkholderia cenocepacia* 891_BCEN | NZ_JUPC00000000 |
| 24 | *Burkholderia cenocepacia* 528_BCEN | NZ_JVDN00000000 |
| 25 | *Burkholderia cenocepacia* FDAARGOS_82 | JTAZ01000001 |

| **Supplementary Table 5**  Annotation of the ORFs of novel genomic island BcenGI15 of *Burkholderia cenocepacia* IPCU-A (Contig 81). BLASTp analysis of ORFs of BcenGI15 along with identified conserved domain, COG class and Pfam family with their predicted putative functions. | | | | | | |  |  |  |  |  |  |  |  |  |
| --- | --- | --- | --- | --- | --- | --- | --- | --- | --- | --- | --- | --- | --- | --- | --- |
|  | | | | | |  | |  |  |  |  |  |  |  |  |
|  |  |  | |  |  |  | |  |  |  |  |  |  |  |  |
| **NCBI Locus Tag** | **BcenGI15 ORFs (RAST)** | **Start** | **Stop** | **Protein length** | **Putative function** | **COG/CDD/pfam** | | **Predicted Functions based on conserved domian, COG and pfam family** |  |  |  |  |  |  |  |
| UE99_30855 | BcenGI15 ORF1 | 12804 | 12884 |  | tRNA-Leu-TAG | - | | - |  |  |  |  |  |  |  |
| UE99_30860 | BcenGI15 ORF2 | 12975 | 13520 | 181 | hypothetical protein | pfam12728 | | Helix-turn-helix domain HTH_17 |  |  |  |  |  |  |  |
| UE99_30865 | BcenGI15 ORF3 | 14570 | 13656 | 304 | hypothetical protein | - | | - |  |  |  |  |  |  |  |
|  | BcenGI15 ORF4 | 15658 | 14636 | 340 | methyltransferase | - | | - |  |  |  |  |  |  |  |
|  | BcenGI15 ORF5 | 15981 | 15847 | 44 | hypothetical protein | - | | - |  |  |  |  |  |  |  |
| UE99_30870 | BcenGI15 ORF6 | 16461 | 16123 | 112 | hypothetical protein | - | | - |  |  |  |  |  |  |  |
|  | BcenGI15 ORF7 | 16843 | 16541 | 100 | hypothetical protein | - | | - |  |  |  |  |  |  |  |
| UE99_30875 | BcenGI15 ORF8 | 17887 | 18249 | 120 | hypothetical protein | - | | - |  |  |  |  |  |  |  |
|  | BcenGI15 ORF9 | 18440 | 18315 | 41 | hypothetical protein | - | | - |  |  |  |  |  |  |  |
| UE99_33880 | BcenGI15 ORF10 | 19162 | 19818 | 218 | hypothetical protein | - | | - |  |  |  |  |  |  |  |
| UE99_30885 | BcenGI15 ORF11 | 19924 | 20160 | 78 | hypothetical protein | - | | - |  |  |  |  |  |  |  |
| UE_30890 | BcenGI15 ORF12 | 20322 | 21155 | 277 | hypothetical protein | - | | - |  |  |  |  |  |  |  |
|  | BcenGI15 ORF13 | 21231 | 21683 | 150 | Outer membrane lipoprotein | COG3133 | | Outer mebrane superfamily,cell envolope Biogenesis and outer membrane |  |  |  |  |  |  |  |
| UE_30895 | BcenGI15 ORF14 | 21839 | 22201 | 120 | hypothetical protein | - | | - |  |  |  |  |  |  |  |
| UE_30900 | BcenGI15 ORF15 | 22866 | 22198 | 222 | hypothetical protein | - | | - |  |  |  |  |  |  |  |
| UE_30905 | BcenGI15 ORF16 | 23055 | 23525 | 156 | hypothetical protein | - | | - |  |  |  |  |  |  |  |
| UE_30910 | BcenGI15 ORF17 | 23512 | 23703 | 63 | hypothetical protein | - | | - |  |  |  |  |  |  |  |
| UE_30915 | BcenGI15 ORF18 | 23700 | 23918 | 72 | hypothetical protein | - | | - |  |  |  |  |  |  |  |
| UE_30920 | BcenGI15 ORF19 | 24357 | 23986 | 123 | hypothetical protein | - | | - |  |  |  |  |  |  |  |
|  | BcenGI15 ORF20 | 24519 | 24674 | 51 | hypothetical protein | - | | - |  |  |  |  |  |  |  |
| UE_30925 | BcenGI15 ORF21 | 24991 | 24794 | 65 | hypothetical protein | - | | - |  |  |  |  |  |  |  |
| UE_30930 | BcenGI15 ORF22 | 25652 | 25155 | 165 | hypothetical protein | - | | - |  |  |  |  |  |  |  |
| UE_30935 | BcenGI15 ORF23 | 25905 | 26861 | 318 | hypothetical protein | pfam07202 | | T-complex protein 10 C-terminus |  |  |  |  |  |  |  |
| UE_30940 |  |  |  |  |  | - | | - |  |  |  |  |  |  |  |
| UE99_30945 | BcenGI15 ORF24 | 27020 | 28663 | 547 | hypothetical protein | - | | - |  |  |  |  |  |  |  |
| UE99_30950 | BcenGI15 ORF25 | 28986 | 29681 | 231 | hypothetical protein | - | | - |  |  |  |  |  |  |  |
|  | BcenGI15 ORF26 | 29899 | 30825 | 308 | hypothetical protein | - | | - |  |  |  |  |  |  |  |
|  | BcenGI15 ORF27 | 30947 | 32023 | 358 | hypothetical protein | - | | - |  |  |  |  |  |  |  |
|  | BcenGI15 ORF28 | 32706 | 32900 | 64 | hypothetical protein | - | | - |  |  |  |  |  |  |  |
| UE99_30955 | BcenGI15 ORF29 | 32912 | 33748 | 836 | hypothetical protein | - | | - |  |  |  |  |  |  |  |
|  | BcenGI15 ORF30 | 33959 | 35107 | 382 | hypothetical protein | - | | - |  |  |  |  |  |  |  |
| UE99_30960 | BcenGI15 ORF31 | 35465 | 36082 | 205 | hypothetical protein | - | | - |  |  |  |  |  |  |  |
|  | BcenGI15 ORF32 | 36151 | 36441 | 96 | hypothetical protein | - | | - |  |  |  |  |  |  |  |
|  | BcenGI15 ORF33 | 36610 | 36978 | 122 | hypothetical protein | - | | - |  |  |  |  |  |  |  |
|  | BcenGI15 ORF34 | 37021 | 37347 | 108 | hypothetical protein | - | | - |  |  |  |  |  |  |  |
| UE99_30965 | BcenGI15 ORF35 | 37756 | 38358 | 200 | hypothetical protein | - | | DNA polymerase III subunits gamma and tau |  |  |  |  |  |  |  |
|  | BcenGI15 ORF36 | 38614 | 38958 | 114 | hypothetical protein | pfam00067 | | Cytochrome P450 |  |  |  |  |  |  |  |
| UE99_30970 | BcenGI15 ORF37 | 38955 | 39419 | 154 | hypothetical protein | - | | SGNH hydrolase |  |  |  |  |  |  |  |
| UE99_30975 | BcenGI15 ORF38 | 40077 | 39457 | 206 | hypothetical protein | - | | - |  |  |  |  |  |  |  |
| UE99_30980 | BcenGI15 ORF39 | 40628 | 40182 | 148 | hypothetical protein | - | | - |  |  |  |  |  |  |  |
| UE99_30985 | BcenGI15 ORF40 | 40805 | 41311 | 168 | hypothetical protein | - | | - |  |  |  |  |  |  |  |
| UE99_30990 | BcenGI15 ORF41 | 41436 | 42464 | 342 | Type IV pilin PilA | pfam00114 | | Pilin A (bacterial filament) |  |  |  |  |  |  |  |
|  | BcenGI15 ORF42 | 42945 | 42832 | 37 | hypothetical protein | - | | - |  |  |  |  |  |  |  |
|  | BcenGI15 ORF43 | 43114 | 43233 | 39 | hypothetical protein | - | | - |  |  |  |  |  |  |  |
|  | BcenGI15 ORF44 | 43857 | 43519 | 112 | hypothetical protein | - | | - |  |  |  |  |  |  |  |
| UE99_30995 | BcenGI15 ORF45 | 44090 | 44446 | 118 | hypothetical protein | - | | - |  |  |  |  |  |  |  |
|  | BcenGI15 ORF46 | 44482 | 44874 | 130 | hypothetical protein | - | | - |  |  |  |  |  |  |  |
|  | BcenGI15 ORF47 | 45170 | 44940 | 76 | hypothetical protein | - | | - |  |  |  |  |  |  |  |
|  | BcenGI15 ORF48 | 47012 | 45291 | 573 | Autotransporter adhesin | pfam03895 | | YadA-like C-terminal region |  |  |  |  |  |  |  |
|  | BcenGI15 ORF49 | 47602 | 47057 | 181 | hypothetical protein | pfam08805 | | PilS N terminal |  |  |  |  |  |  |  |
| UE99_31000 | BcenGI15 ORF50 | 49080 | 47755 | 441 | surface protein | cd12820 | | YadA-like, left-handed beta-roll(lbr like family ) |  |  |  |  |  |  |  |
| UE99_31005 | BcenGI15 ORF51 | 49373 | 49645 | 90 | YefM protein (antitoxin to YoeB) | pfam02604 | | Antitoxin Phd_YefM, type II toxin-antitoxin system,PhdYeFM_antitox |  |  |  |  |  |  |  |
| UE99_31010 | BcenGI15 ORF52 | 49645 | 49980 |  | conserved domain protein | COG3668 | | ParE ,Plasmid stabilization system protein |  |  |  |  |  |  |  |
| UE99_31015 | BcenGI15 ORF53 | 50107 | 52401 | 764 | hypothetical protein | - | | - |  |  |  |  |  |  |  |
|  | BcenGI15 ORF54 | 52543 | 52758 | 71 | hypothetical protein | - | | - |  |  |  |  |  |  |  |
| UE99_31020 | BcenGI15 ORF55 | 53536 | 52814 | 240 | Adenine-specific methyltransferase | pfam01555 | | N6_N4_Mtase (Adinine methyltransferase) |  |  |  |  |  |  |  |
| UE99_31025 | BcenGI15 ORF56 | 54539 | 54018 | 173 | hypothetical protein |  | |  |  |  |  |  |  |  |  |
| UE99_31030 | BcenGI15 ORF57 | 54768 | 55493 | 241 | hypothetical protein | COG4942 | | Membrane-bound metallopeptidase [Cell division and chromosome partitioning] |  |  |  |  |  |  | **Column1** |
| UE99_31055 | BcenGI15 ORF58 | 55654 | 56061 | 135 | hypothetical protein | - | | - |  |  |  |  |  |  |  |
| UE99_31040 | BcenGI15 ORF59 | 56118 | 56498 | 126 | hypothetical protein | - | | - |  |  |  |  |  |  |  |
|  | BcenGI15 ORF60 | 56603 | 56487 | 38 | hypothetical protein | - | | - |  |  |  |  |  |  |  |
|  | BcenGI15 ORF61 | 56682 | 56990 | 102 | hypothetical protein | - | | - |  |  |  |  |  |  |  |
| UE99_31045 | BcenGI15 ORF62 | 57128 | 57817 | 299 | hypothetical protein | - | | - |  |  |  |  |  |  |  |
|  | BcenGI15 ORF63 | 58310 | 58552 | 80 | hypothetical protein | - | | - |  |  |  |  |  |  |  |
| UE99_31050 | BcenGI15 ORF64 | 58645 | 59631 | 328 | hypothetical protein | COG3249 | | Uncharacterized protein conserved in bacteria [Function unknown] |  |  |  |  |  |  |  |
| UE99_31055 | BcenGI15 ORF65 | 59803 | 59994 | 63 | hypothetical protein | - | | - |  |  |  |  |  |  |  |
| UE99_31060 | - | - | - | - | - | - | | - |  |  |  |  |  |  |  |
|  | BcenGI15 ORF66 | 60167 | 61525 | 452 | ATP-dependent protease domain protein | cd00009 | | The AAA+ (ATPases Associated with a wide variety of cellular Activities) |  |  |  |  |  |  |  |
|  | BcenGI15 ORF67 | 61755 | 61898 | 47 | hypothetical protein | - | | - |  |  |  |  |  |  |  |
| UE99_31065 | BcenGI15 ORF68 | 61923 | 62243 | 106 | hypothetical protein | - | | - |  |  |  |  |  |  |  |
|  | BcenGI15 ORF69 | 62450 | 63328 | 292 | hypothetical protein | - | | - |  |  |  |  |  |  |  |
| UE99_31070 | BcenGI15 ORF70 | 63381 | 64064 | 227 | hypothetical protein | - | | - |  |  |  |  |  |  |  |
|  | BcenGI15 ORF71 | 64213 | 65679 | 448 | hypothetical protein | - | | - |  |  |  |  |  |  |  |
| UE99_31075 | BcenGI15 ORF72 | 65816 | 66163 | 115 | hypothetical protein | - | | - |  |  |  |  |  |  |  |
| UE99_31080 | BcenGI15 ORF73 | 66261 | 66740 | 159 | hypothetical protein | - | | - |  |  |  |  |  |  |  |
| UE99_31085 | BcenGI15 ORF74 | 66924 | 67136 | 70 | hypothetical protein | - | | - |  |  |  |  |  |  |  |
|  | BcenGI15 ORF75 | 67508 | 67636 | 42 | hypothetical protein | - | | - |  |  |  |  |  |  |  |
| UE99_31090 | BcenGI15 ORF76 | 67674 | 68219 | 181 | type III restriction system methylase | - | | - |  |  |  |  |  |  |  |
|  | BcenGI15 ORF77 | 68407 | 68288 | 39 | hypothetical protein | - | | - |  |  |  |  |  |  |  |
|  | BcenGI15 ORF78 | 68601 | 68855 | 84 | hypothetical protein | - | | - |  |  |  |  |  |  |  |
|  | BcenGI15 ORF79 | 69182 | 69349 | 55 | hypothetical protein | - | | - |  |  |  |  |  |  |  |
|  | BcenGI15 ORF80 | 69648 | 70340 | 230 | hypothetical protein | cd00204 | | ankyrin repeats |  |  |  |  |  |  |  |
| UE99_31095 | BcenGI15 ORF81 | 70496 | 71035 | 179 | Phage protein | cd06127 | | DEDDh 3'-5' exonuclease domain family |  |  |  |  |  |  |  |
| UE99_31100 | BcenGI15 ORF82 | 72260 | 71088 | 390 | hypothetical protein | - | | - |  |  |  |  |  |  |  |
|  | BcenGI15 ORF83 | 72469 | 72723 | 84 | hypothetical protein | - | | - |  |  |  |  |  |  |  |
|  | BcenGI15 ORF84 | 72738 | 73346 | 202 | D-alanyl-D-alanine dipeptidase | COG2173 | | vanY superfamily |  |  |  |  |  |  |  |
|  | BcenGI15 ORF85 | 73303 | 73836 | 177 | hypothetical protein | - | | - |  |  |  |  |  |  |  |
|  | BcenGI15 ORF86 | 73972 | 74166 | 64 | hypothetical protein | - | | - |  |  |  |  |  |  |  |
| UE99_31105 | BcenGI15 ORF87 | 74265 | 74792 | 175 | hypothetical protein | - | | - |  |  |  |  |  |  |  |
| UE99_31110 | BcenGI15 ORF88 | 76367 | 76594 | 75 | hypothetical protein | - | | - |  |  |  |  |  |  |  |
| UE99_31115 | BcenGI15 ORF89 | 77165 | 76890 | 91 | hypothetical protein | - | | - |  |  |  |  |  |  |  |
|  | BcenGI15 ORF90 | 77942 | 77304 | 212 | hypothetical protein | - | | - |  |  |  |  |  |  |  |
| UE99_31120 | BcenGI15 ORF91 | 78793 | 79197 | 134 | hypothetical protein | - | | - |  |  |  |  |  |  |  |
|  | BcenGI15 ORF92 | 79303 | 79959 | 218 | hypothetical protein | cd00204 | | ankyrin repeats |  |  |  |  |  |  |  |
| UE99_31125 | BcenGI15 ORF93 | 80091 | 80474 | 127 | hypothetical protein | - | | - |  |  |  |  |  |  |  |
| UE99_31130 | BcenGI15 ORF94 | 82709 | 81933 | 258 | hypothetical protein | - | | - |  |  |  |  |  |  |  |
| UE99_31135 | BcenGI15 ORF95 | 83116 | 83472 | 118 | hypothetical protein | COG0040 | | ATP phosphoribosyltransferase [Amino acid transport and metabolism] |  |  |  |  |  |  |  |
| UE99_31140 | BcenGI15 ORF96 | 83472 | 87068 | 1198 | Conjugal transfer protein TraA | cd00009 | | ATPases Associated with a wide variety of cellular Activities superfamily |  |  |  |  |  |  |  |
| UE99_31145 | BcenGI15 ORF97 | 87235 | 87981 | 248 | adenine specific DNA methyltransferase (dpnA) | pfam01555 | | N6_N4_Mtase (Adinine methyltransferase) |  |  |  |  |  |  |  |
|  | BcenGI15 ORF98 | 88121 | 88480 | 119 | hypothetical protein | - | | - |  |  |  |  |  |  |  |
| UE99_31150 | BcenGI15 ORF99 | 88485 | 88733 | 82 | hypothetical protein | - | | - |  |  |  |  |  |  |  |
| UE99_31155 | BcenGI15 ORF100 | 88843 | 89793 | 316 | hypothetical protein | - | | - |  |  |  |  |  |  |  |
|  | BcenGI15 ORF101 | 90370 | 90606 | 78 | hypothetical protein | - | | - |  |  |  |  |  |  |  |
| UE99_31160 | BcenGI15 ORF102 | 90668 | 92911 | 747 | IncW plasmid conjugative protein TrwB (TraD homolog) | cd01127 | | Bacterial conjugation protein TrwB |  |  |  |  |  |  |  |
| UE99_31165 | - | - | - | - | - | - | | - |  |  |  |  |  |  |  |
| UE99_31170 | - | - | - | - | - | - | | - |  |  |  |  |  |  |  |
| UE99_31175 | BcenGI15 ORF103 | 93013 | 95133 | 706 | hypothetical protein | - | | - |  |  |  |  |  |  |  |
| UE99_31180 | BcenGI15 ORF104 | 96148 | 95189 | 319 | hypothetical protein | - | | - |  |  |  |  |  |  |  |
| UE99_31185 | BcenGI15 ORF105 | 96274 | 97164 | 296 | hypothetical protein | - | | - |  |  |  |  |  |  |  |
| UE99_31190 | BcenGI15 ORF106 | 97180 | 100656 | 1158 | hypothetical protein | pfam12696 | | TraM recognition site of TraD and TraG |  |  |  |  |  |  |  |
|  | BcenGI15 ORF107 | 101253 | 101681 | 142 | hypothetical protein | - | | - |  |  |  |  |  |  |  |
| UE99_31195 | BcenGI15 ORF108 | 102247 | 103884 | 545 | hypothetical protein | cd00204 | | ankyrin repeats |  |  |  |  |  |  |  |
| UE99_31200 | BcenGI15 ORF109 | 103944 | 105317 | 457 | hypothetical protein | - | | - |  |  |  |  |  |  |  |
| UE99_31205 | BcenGI15 ORF110 | 105656 | 106345 | 229 | hypothetical protein | - | | - |  |  |  |  |  |  |  |
| UE99_31210 | BcenGI15 ORF111 | 106523 | 107191 | 222 | hypothetical protein | cd00204 | | ankyrin repeats |  |  |  |  |  |  |  |
|  | BcenGI15 ORF112 | 107320 | 108408 | 362 | hypothetical protein | - | | - |  |  |  |  |  |  |  |
| UE99_31215 | BcenGI15 ORF113 | 109897 | 108800 | 365 | Antirestriction protein | COG4227 | | Antirestriction protein [DNA replication, recombination, and repair] |  |  |  |  |  |  |  |
|  | BcenGI15 ORF114 | 110032 | 110241 | 69 | hypothetical protein | - | | - |  |  |  |  |  |  |  |
| UE99_31220 | BcenGI15 ORF115 | 110234 | 111055 | 273 | hypothetical protein | COG3672 | | Predicted transglutaminase-like cysteine proteinase |  |  |  |  |  |  |  |
| UE99_31225 | BcenGI15 ORF116 | 111189 | 111422 | 77 | hypothetical protein | - | | - |  |  |  |  |  |  |  |
| UE99_31230 | BcenGI15 ORF117 | 111425 | 111862 | 145 | hypothetical protein | - | | - |  |  |  |  |  |  |  |
| UE99_31235 | BcenGI15 ORF118 | 111865 | 112437 | 190 | hypothetical protein | - | | - |  |  |  |  |  |  |  |
| UE99_31240 | - | - | - | - | - | - | | - |  |  |  |  |  |  |  |
| UE99_31245 | BcenGI15 ORF119 | 114680 | 112503 | 725 | conjugative transfer integral membrane protein TraY | TIGR04346 | | conjugal transfer/type IV secretion protein DotA/TraY |  |  |  |  |  |  |  |
| UE99_31250 | BcenGI15 ORF120 | 114735 | 114926 | 63 | hypothetical protein | - | | - |  |  |  |  |  |  |  |
| - | BcenGI15 ORF121 | 115569 | 115336 | 277 | hypothetical protein | - | | - |  |  |  |  |  |  |  |
| - | BcenGI15 ORF122 | 116729 | 115701 | 342 | Transcriptional regulator | pfam07669 | | Eco57I restriction-modification methylase |  |  |  |  |  |  |  |
| - | BcenGI15 ORF123 | 117263 | 117117 | 48 | hypothetical protein | - | | - |  |  |  |  |  |  |  |
| UE99_31255 | BcenGI15 ORF124 | 118297 | 117683 | 204 | hypothetical protein | - | | - |  |  |  |  |  |  |  |
| UE99_31260 | BcenGI15 ORF125 | 119672 | 118323 | 449 | Shufflon-specific DNA recombinase | cd00796/pfam00589 | | Shufflon-specific DNA recombinase , Phage_integrase family |  |  |  |  |  |  |  |
| UE99_31265 | BcenGI15 ORF126 | 119623 | 119766 | 47 | hypothetical protein | - | | - |  |  |  |  |  |  |  |
|  |  |  |  |  |  |  | |  |  |  |  |  |  |  |  |
